# Disinfectants and one health review: The role of reactive oxygen species in the bactericidal activity of chlorine against *Salmonella*^☆^

**DOI:** 10.1016/j.onehlt.2025.100989

**Published:** 2025-02-06

**Authors:** Mohammed Aljuwayd, Israa Abdullah Malli, Elena G. Olson, Steven C. Ricke, Michael J. Rothrock, Young Min Kwon

**Affiliations:** a Cell and Molecular Biology Program, University of Arkansas, Fayetteville, AR 72701, USA; b College of Medical Applied Sciences, The Northern Border University, Arar 91431, Saudi Arabia; c College of Medicine, King Saud bin Abdulaziz University for Health Sciences, Jeddah 21423, Saudi Arabia; d King Abdullah International Medical Research Center, Jeddah 22384, Saudi Arabia; e Meat Science and Animal Biologics Discovery Program, Department of Animal and Dairy Sciences, University of Wisconsin, Madison, WI 53706, USA; f United States Department of Agriculture, Agricultural Research Service, Athens, GA 30605, USA; g Department of Poultry Science, University of Arkansas System, Division of Agriculture, Fayetteville, AR 72701, USA

**Keywords:** Salmonella, Disinfectants, Food safety, Reactive oxygen species, Chlorine, Poultry

## Abstract

*Salmonella* are among the most common foodborne pathogens in humans, and they are associated with mild to severe diseases commonly referred to as salmonellosis. The genus resides in various animals' intestinal tracts, including humans. It is one of the most diverse genera of bacteria, including over 2500 serovars. Consumption of poultry products contaminated with *Salmonella* is a significant source of disease transmission in humans. Because of this food safety concern, the poultry industry and governments spend billions of dollars on *Salmonella* containment methods. However, a completely effective strategy is yet to be established. Chlorine has been commonly used as a disinfectant in the poultry industry. In humans, antibiotic therapy is the primary means for managing *Salmonella* infection. However, widespread use of both compounds at sub-inhibitory concentrations has allowed resistant strains to emerge and rapidly spread globally. Both antimicrobial compounds involve generating reactive oxygen species (ROS) as a bactericidal mechanism of action. However, ROS generation and its association with bacterial survival and growth inhibition have not been widely explored. Thus, a better understanding of ROS generation during antimicrobial treatments may help devise better *Salmonella* containment strategies.

## 1 The role of chickens in *Salmonella enterica* infections and their impact on human health

**Fig. 1:**
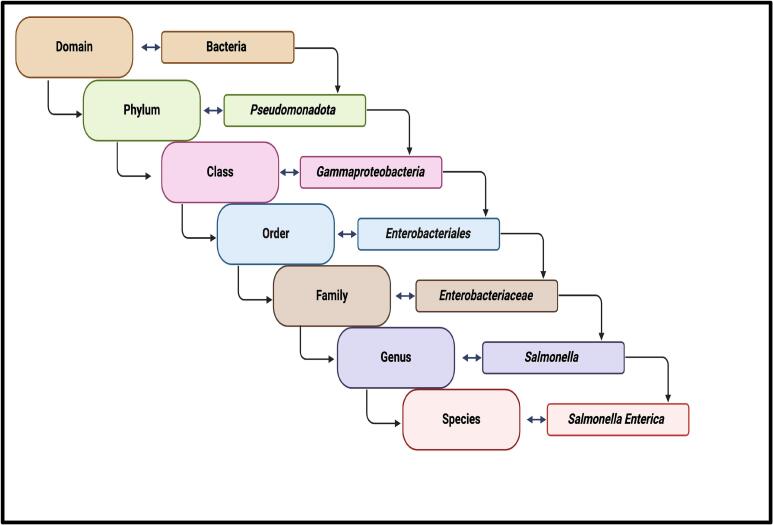
The taxonomy of *Salmonella* species. Figure generated by BioRender (Malli, I. (7-9-2024), BioRender.com/FQ271HJXH2, accessed on 9 July 2024).

*Salmonella* is a Gram-negative, rod-shaped, motile, hydrogen sulfide-generating, acid-labile, facultative intracellular microorganism that causes food poisoning [1,2]. *Salmonella* species are motile enterobacteria within the *Enterobacteriaceae* family (classification scheme outlined in Fig. 1). *Salmonella* have peritrichous flagella and hair-like appendages that protrude from cells around their cell surface, which enables motility [3]. *Salmonella* spp. are described as facultative anaerobic organisms and generate ATP by aerobic respiration in the presence of oxygen but are also capable of fermentation, obtaining energy from oxidation and reduction reactions of organic compounds [4,5]. Approximately 2500 distinct serovars of *Salmonella* have been identified. *Salmonella* genus is broadly grouped into two species: *Salmonella bongori* and *Salmonella enterica. Salmonella enterica* contains six subspecies: *enterica*, *arizonae*, *diarizonae*, *salamae*, *indica*, and *houtenae* [2,6]. *Salmonella* subspecies consist of numerous serovars. *S*. *enterica* subspecies *enterica* encompasses a wide array of serovars, and several are foodborne pathogens. Some of the more prevalent and pathogenic *Salmonella enterica* serovars include Enteritidis, Typhimurium, Javiana, and Newport [7,8].

*Salmonella* species often cause cross-infections between humans and livestock. Animals are notable carriers of *Salmonella*, with the bacteria typically residing in the intestines and being shed through feces [2]. Humans frequently become infected by consuming contaminated food or water [9]. Contamination can occur throughout the food production chain, producing infected final products [[10], [11], [12], [13]]. Ingesting poultry meat and eggs contaminated with *Salmonella* can lead to infections, mainly affecting the intestinal tract and causing inflammatory diarrhea [14,15].

**Fig. 2:**
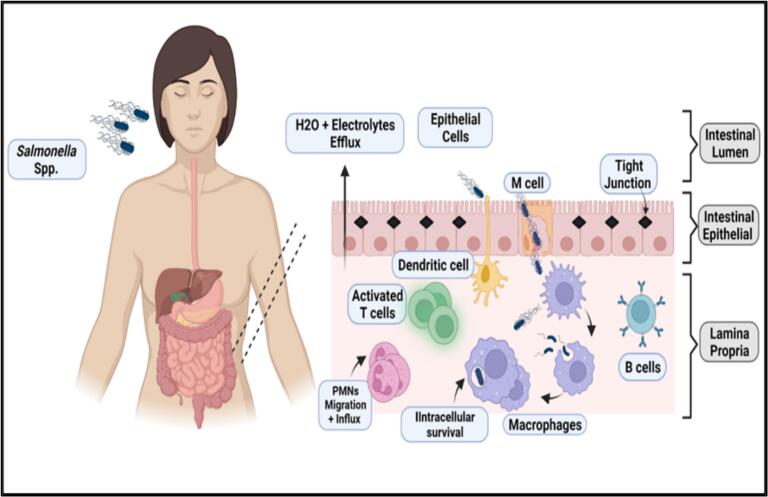
*Salmonella* pathogenesis inside the human gut. *Salmonella* attaches to the intestinal epithelium cells through adhesins molecules. The invasion of bacteria occurs next, and engulfment is mediated by virulence adhesion proteins, which will disturb the gap cellular junction. Bacterial cells may also be taken up directly by dendritic cells from the intestinal lumen to be released in the lamina propria. After macrophages uptake, *Salmonella* survives and multiplies intracellularly within the cytoplasm; thus, contaminated cells may spread via the lymph and bloodstream. Figure generated by BioRender (Malli, I. (7-9-2024), BioRender.com/GA271HL2LC, accessed on 9 July 2024).

*Salmonella enterica* can cause nontyphoidal *Salmonella* infections in humans and other vertebrates [15]. *S. enterica* can colonize animals, humans, plants, and environmental locations. This zoonotic infection can be transferred from human to human or animal to human [[16], [17]]. The bacteria usually invade the gastrointestinal tract (GIT), causing salmonellosis, a significant threat that can lead to illness and death in humans and animals [4]. Upon ingestion, *S. enterica* invades the intestinal epithelium, causing neutrophilic gastroenteritis or spreading to systemic locations, resulting in sepsis [1,7]. These bacteria are considered intracellular pathogens, capable of invading, growing and reproducing inside host cells, allowing for intrinsic antimicrobial resistance and, in rare cases, persistent colonization [[18], [19]]. Fig. 2 illustrates *Salmonella* pathogenesis inside the human gut. In immunocompromised individuals, *Salmonella* may produce significant localized infections [9]. Serovars Typhi and Paratyphi can cause typhoidal *Salmonella* and enteric fever [20]. Typhoidal *Salmonella* is host-specific, generally found in humans, and is invasive. The non-invasive serotype leads to foodborne infections and typhoid fever, which can be transmitted from one person to another. The invasive typhoidal serotypes secrete endotoxins that may result in life-threatening hypovolemic or septic infections [21,22].

Salmonellosis is a symptomatic infection caused by non-typhoid *Salmonella* serotypes, which include symptoms such as abdominal cramps, vomiting, diarrhea, fever, and dehydration [[23], [24], [25], [26], [27], [28]]. *Salmonella* infection cannot be clinically separated from illnesses caused by other intestinal bacterial infections [25]. Illness generally develops after consuming a sufficient infectious dose of bacteria in contaminated food or drink, with an incubation period of 6 to 72 h, depending on host susceptibility and initial inoculum size. This process involves delivering toxins to GIT tissues produced by *Salmonella* oxidative activities. Although non-typhoidal serovars often cause self-limiting diarrhea in immunocompetent hosts, untreated cases can result in fatalities. Factors such as patient age and alterations of the endogenous GIT microbiota after antimicrobial therapy or surgical intervention might predispose the host to severe and invasive *Salmonella* colonization [29]. Immunocompromised patients are more likely to acquire localized infections such as meningitis, septic arthritis, osteomyelitis, cholangitis, and pneumonia. For instance, the development of infectious endarteritis, particularly of the abdominal aorta, can result from *Salmonella* bacteremia in adults [27,30].

The host carrier state is another clinical consequence of *Salmonella* infections, applicable to both typhoid and non-typhoidal infections in all hosts humans and animals. It is defined as chronic GIT colonization, developing approximately 8 to 10 weeks after the original infection. Biofilm formation is often a physiological feature contributing to long-term persistence [26,31]. Host carriers of *Salmonella* can contribute to the spread of the illness, which is especially relevant for food workers. Understanding factors associated with *Salmonella* carriers, such as immune response and antimicrobial effectiveness, can aid in creating appropriate preventative and therapeutic intervention strategies [3]. Treatment options for salmonellosis typically involve supportive care, such as rehydration and electrolyte replacement. In severe cases, particularly for invasive infections, antibiotics such as fluoroquinolones or third-generation cephalosporins may be prescribed. However, the increasing antibiotic resistance among *Salmonella* strains is a growing concern, necessitating careful use of these medications.

Recent epidemiological statistics reveal that *Salmonella* infections continue to pose a significant public health concern globally. This is consistent with observed worldwide trends indicating a rising incidence of *Salmonella*-related illnesses [32] These increases are attributed to multiple factors, including the contamination of food products across the supply chain, particularly poultry and other animal-based foods, which are common vehicles for foodborne transmission [33]. Due to the high prevalence of *Salmonella* in broilers and its association with human salmonellosis and persistence in outbreaks, there has been increased emphasis on reducing *Salmonella* in chicken farms and final products [34]. This includes monitoring and regulating antibiotic use, enhancing farm hygiene, and developing new vaccines and treatment protocols to ensure food safety and public health. Prevention strategies include farm biosecurity measures, poultry vaccination programs, and stringent food-processing hygiene practices [34]. However, pathogens such as *Salmonella* can persist in the environment because it can develop resistance when subjected to various preventive pressures [35,36]. Historically, antibiotics have been used to combat pathogens in animal production. However, improper antibiotic use may pose health risks to consumers. As a result, it has become necessary to investigate novel compounds and approaches to combat poultry-associated pathogens such as *Salmonella* [[35], [36], [37]]. Several active chemical compounds combat pathogens on non-living surfaces using disinfectants. For example, water used in poultry farms and during processing usually includes disinfectants to avoid the proliferation of pathogens among poultry flocks, processing facilities, and, thus, the final product [[36], [37], [38], [39]].

This review will specifically focus on chlorine and its various derivatives and their use as disinfectants in the poultry industry. It is also critical to understand the antimicrobial mechanisms associated with chlorine-based disinfection and decontamination strategies. Recent research indicates that manipulating reactive oxygen species (ROS) levels can significantly enhance the efficacy of antimicrobial treatments such as chlorine-based compounds. Thus, the remainder of the review will explore the role that ROS may play with chlorine antimicrobial activities.

## 2 Application of chlorine in poultry production for pathogen mitigation

Chlorine-containing compounds have been used since 1868 and are still generally utilized due to their low cost, ease of implementation, and high efficacy [40,41]. These include active chlorine compounds such as chlorine, hypochlorite, chloramines, chlorine dioxide, sodium dichloro isocyanurate, and trichloroisocyanuric acid [41]. Several of these chlorine-based compounds have been used in poultry production over the years [39,42,43]. Treatment of drinking water in poultry houses involves inclusion of sanitizers such as chlorine-based products to the water [39]. Application of chlorine in the U.S. has also been employed for table eggs as they exit the egg washer [42]. However, efficacy in these different systems can be variable depending on factors such as the raw water quality [44].

Variations of chlorine-based compounds such as sodium hypochlorite have been used as poultry processing decontamination agents [45]. Sodium hypochlorite has been reported to be a potentially effective poultry disinfectant [45]. When added to water, chlorine reacts with the hydrogen and oxygen of water molecules, forming hydrochloric acid and hypochlorous acid. Hypochlorous acid is then dissociated to produce hypochlorite and hydrogen ions. Hypochlorous acid and hypochlorite account for “free chlorine” in a solution and are the main compounds responsible for the antimicrobial action of chlorine [43,45]. The free chlorine can affect bacterial cells in different ways. First, the free chlorine compounds can disrupt the bacterial cell wall. Secondly, the free chlorine can interact with the cell nucleic material and enzymes, inhibiting their normal processes.

Chlorine-containing compounds have been used for water treatment as well as decontamination of raw poultry products [45,46]. In addition, chlorine-containing compounds such as acidified sodium chlorite have been reported to reduce foodborne pathogens such as *Salmonella* [45]. Other chlorine compounds have also been examined for use in poultry processing plants [45]. For example, studies have shown that chlorine dioxide causes bacterial inactivation by disrupting the bacterial dehydrogenase enzymes, inhibiting protein synthesis [[46], [47], [48]]. However, the degree to which protein synthesis is inhibited is closely connected to the initial concentration of chlorine dioxide supplied because regulator proteins can accumulate to protect the cell from damage [47,48]. In addition, chlorine penetration into cell walls can be reduced in the presence of the lower temperatures associated with poultry carcass chillers [46].

Due to the development of toxic byproducts and other possible risks, legislation on chlorine-containing chemicals in the food sector is becoming increasingly rigorous. The use of chlorine-containing antimicrobials in chicken processing to treat *Salmonella* species may be a significant concern for promotion of antimicrobial resistance [49]. Although chlorine may decrease *Salmonella* prevalence, lower concentrations can enhance the selection of chlorine-resistant variants of *Salmonella*. Therefore, chlorination may present significant challenges for future bacterial treatment due to the development of chlorine resistance [50]. Research that explored *Salmonella*'s resistance to hypochlorous acid [51] is a primary example of this. Hypochlorous acid-resistant strains were shown to alter dehydrogenase activity, resulting in lower quantities of oxygen and hydroxyl radicals, the molecules primarily responsible for antibacterial effects [35,52]. In chilling tanks, if *Salmonella* survives, they can develop resistance genes, potentially leading to mutations and the formation of multidrug-resistant (MDR) strains. When MDR *Salmonella* infects humans, this can result in a challenge for clinical treatment.

Environmental, host-derived stimuli and intracellular responses can promote mutagenesis, potentially resulting in multi-drug resistance in food-borne pathogens. ROS molecules are among the metabolic processes that interact with sanitizers and antibiotic exposure. ROS-induced mutagenesis can induce antibiotic resistance in several foodborne pathogens [[52], [53], [54]]. An occurrence of mutations, followed by erroneous repair mechanisms, can lead to genetic alterations such as rifampin resistance associated with mutations in the *rpoB* gene [55]. These findings further challenge clinical treatments by limiting the range of antibiotics that may be effectively administered in human applications [53]. An in-depth understanding of the organism's properties and implications is necessary to elucidate the *Salmonella* effect. This could in turn, impact *Salmonella* risk assessment models for the poultry production chain [56]. Determining if ROS-induced mutations are strain- or serovar-specific upon exposure to sublethal chlorine-based disinfectants is crucial. The ability to deal with oxygen toxicity may in part due to physiological state of *Salmonella* such as whether is growing aerobically or anaerobically and the subsequent impact on iron metabolism [57,58].

## 3 *Salmonella* resistance to disinfectants in poultry production and ROS implications

**Fig. 3:**
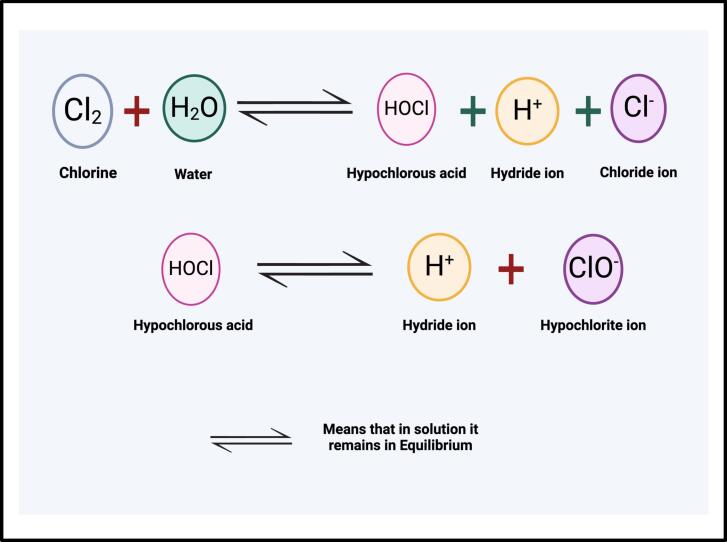
Chlorine reaction and killing mechanism. Chlorine reacts in water to form hypochlorous acid, which can then dissociate into hydrogen and hypochlorite ions. This reaction is critical, as the disinfecting power of HOCl, hypochlorous acid, is significantly greater than that of ClO-, hypochlorite ion. Figure generated by BioRender (Malli, I. (7-9-2024), BioRender.com/YA271HIZHO, accessed on 9 July 2024).

Reactive oxygen species (ROS) are cell signaling molecules that contain at least one oxygen atom and unpaired electron. ROS are molecules that are generated during normal metabolic processes and in response to stress. They include superoxide anions (O_2_-), hydrogen peroxide (H_2_O_2_), and hydroxyl radicals (OH-). These molecules are highly reactive and can cause damage to DNA, proteins, and lipids within cells, leading to cell death if not neutralized or controlled effectively. ROS can be produced endogenously by bacteria as a byproduct of aerobic respiration or found exogenously in the host environment [59]. Superoxide and hydrogen peroxide, two of the most studied forms of ROS, are continually created endogenously by the oxidation of O_2_ by various aerobic and non-aerobic respiratory flavoproteins [59]. Fig. 3 illustrates chlorine reaction and killing mechanism.

The oxidative stress response has been well-studied in *Salmonella,* including the production of neutralizing enzymes to prevent cellular damage, such as peroxidases, catalases, and glutathione reductase, by converting them into less harmful molecules [60]. Resistance to hydrogen peroxide can occur in *Salmonella* and can involve numerous pathways including iron homoeostasis, DNA repair, Fe—S cluster biosynthesis and hydrogen peroxide scavenging systems among others [61]. Thus, tolerance to hydrogen peroxide during the oxidative stress response may not significantly impact organisms exposed to various antiseptics and disinfectants [62].

Chlorine-based water disinfection can increase ROS levels and DNA release into an environment which in turn, can disseminate microbial genes associated with antibiotic resistance [63]. Microorganisms can acquire antimicrobial resistance in several ways [64]. Some of these ways are accelerated mutagenesis in hypermutator lines, the transfer of resistance genes by horizontal means, and the specific selection of clearly resistant variations within the population. In addition, given their distinct cellular structure and physiology, bacteria may respond differently to disinfectants and antibiotics. Historically, microbiological sensitivity to disinfectants has been classified based on these distinctions [[65], [66], [67]]. An organism's resistance may be innate or acquired via mutation, the acquisition of plasmids, extrachromosomal DNA, or transposons or by adopting a phenotypically tolerant physiology to environmental shifts [68,69]. For example, Gram-negative bacteria, bacterial spores, and mycobacteria can exhibit intrinsic resistance [68,70]. However, acquired resistance can occur in *Salmonella* that may be obtained through horizontal gene transfer [70].

## 4 Understating ROS and pathogen resistance to antibiotics in human application of antimicrobials

**Fig. 4:**
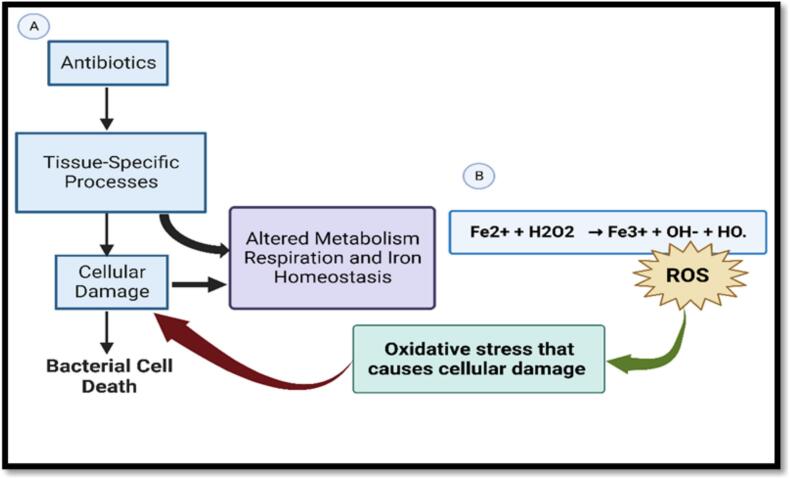
Bactericidal antibiotics promote the generation of toxic reactive species: (A) Bactericidal antibiotics of different classes can induce cell death by interfering with their primary targets and corrupting target-specific processes, resulting in lethal cellular damage. Target-specific interactions trigger stress responses that induce redox-related physiological alterations, resulting in the formation of toxic reactive species, including ROS, which further contribute to cellular damage and death. (B) Illustration of Fenton's reaction as a significant source of OH. The ferrous and/or ferric cations catalytically decompose hydrogen peroxide to produce potent oxidizing agents that can destroy many inorganic and organic compounds. Figure generated by BioRender (Malli, I. (7-9-2024), BioRender.com/NY271HISKM, accessed on 9 July 2024).

The development of antibiotic resistance in foodborne pathogens such as *Salmonella* can further complicate treatment post-consumption [70,71]. This coupled with the ability of pathogens to overcome host defenses and persist for long periods of time can have major implications on treatment approaches [71,72]. Antimicrobials can inhibit DNA and RNA synthesis and the production of proteins and cell wall components. The mode of action of antimicrobials is commonly categorized based on their interaction with cellular targets [73]. Antimicrobial-mediated bacterial death is frequently attributed to DNA strand breaks, inhibition of RNA synthesis, destruction of cell wall synthesis, ribosome binding, and defective translation of prokaryotic genes [74]. Utilization of sub-lethal concentrations of antibiotics has been shown to induce mutagenesis in bacteria, leading to increased minimum inhibitory concentrations for various bactericidal drugs. Antimicrobial compounds such as hydrogen peroxide can be encountered in varying concentrations when produced as a defense agent by bacteria, plants, and animals [75]. Quinolones, used for treating *Salmonella*-associated infections, can accelerate the development of antibiotic resistance by inducing *recA*-facilitated processes and SOS-autonomous recombination. Quinolones, β-lactams, and aminoglycosides can cause microorganisms to generate low levels of ROS, leading to DNA mutations and prompting the error-prone SOS response [53,63,64]. This mutagenesis is associated with increased ROS, revealing another mechanism by which low doses of antibiotics can induce multi-drug resistance, highlighting additional implications for antibiotic use. Fig. 4 illustrates the mechanism for the formation of hydroxyl radicals [53,54,75].

Host factors, including immune components such as phagocytes, are crucial in pathogen destruction. Although the mechanism is not fully understood, phagocytes produce exogenous ROS in response to pathogen recognition [[76], [77], [78], [79], [80], [81]]. Numerous environmental and host responses can lead to ROS generation and repair mechanisms are employed by organisms to combat oxidative damage [81]. For example, transcription factors in *S. enterica* can be activated by directly oxidizing their sensor proteins, thereby activating regulons that govern antioxidant defense genes, such as superoxide dismutase, ferric uptake regulator, ferritin, and bacterioferritin [[81], [82], [83], [84]]. Iron homeostasis is essential for mitigating oxidative damage. Consequently, iron-sensing transcriptional repressors such as ferric uptake regulators in *Salmonella* species maintain oxidation-reduction homeostasis by controlling the expression of genes encoding iron acquisition systems and iron-dependent enzymes [85].

Ultimately, the interplay between ROS-induced mutagenesis and resistance in foodborne pathogens such as *Salmonella* presents significant challenges to public health. Understanding the mechanisms by which sub-lethal concentrations of disinfectants and the host environment contribute to this resistance is crucial for developing effective treatment strategies. Enhanced knowledge of microbial responses to oxidative stress and the role of cellular metabolism can lead to better clinical practices and the development of novel therapeutics to combat antibiotic-resistant infections. Addressing these issues is essential for maintaining the efficacy of disinfectants such as chlorine and safeguarding human health.

## 5 Strategies to overcome ROS-induced antibacterial resistance in *Salmonella*

Some strains of *Salmonella* may acquire resistance to disinfectants via modifying cell membranes, generating protective biofilms, or synthesizing enzymes that counteract the disinfectant's efficacy. Environments such as hatcheries, cages, and feed can potentially *Salmonella* to sub-lethal doses of disinfectants, making resistance a significant challenge. *Salmonella* biofilms build up on metal and plastic surfaces, which are common in food processing environments [86]. This protects *Salmonella* from ROS produced by chlorine-based disinfectants, making it more difficult for the disinfectants to interact with the bacteria and ultimately kill them. Furthermore, biofilms may require greater disinfectant doses or extended exposure, which may unintentionally promote resistant bacteria. Where large numbers of broilers with high density occupancy share the same floor covered with litter material as well as the same environmental conditions it is likely that biofilms will form and lead to ineffectiveness of disinfectants when surface applied [87]. If these biofilms are present, they could neutralize disinfectants such as chlorine before they come in contact with *Salmonella* cells. Using mechanistically different types of disinfectants to prevent bacteria from adapting to a single disinfectant type, could also improve the efficacy of hygiene practices to prevent biofilm formation, along with applying disinfectants at appropriate rates and durations to prevent the formation of resistant *Salmonella* strains.

## 6 Endogenous ROS production

Increasing microbial ROS production can potentiate killing by antibiotics and oxidants. Targeting bacterial mechanisms that promote ROS damage may help to improve antimicrobial therapy. Most antimicrobials can produce ROS by lysed bacteria, where the generation of ROS is commonly associated with disruption of the TCA cycle, low NADH levels, and increased ROS formation [54,81,86]. For example, quinolones act on DNA replication by binding DNA gyrase and DNA, causing double-strand breaks, and eventually cell death. Thus, synergistic application to promote gyrase inhibitors can stimulate the generation of high levels of ROS that contribute to cellular death [86,87].

Enhanced ROS produced by NADPH oxidase (NOX) can improve pathogen clearance by activating a range of innate and adaptive processes in the host cell. NOX-derived ROS are required for autophagy to boost antimicrobial activity. During an infection, neutrophils and phagocytes may activate a second antimicrobial mechanism known as neutrophil extracellular traps (NET) [88,89]. ROS generation is required for NET formation [90], which is hindered in NOX-deficient neutrophils [89]. A broad-spectrum antibacterial has been utilized to improve NET-mediated lysis of *Staphylococcus aureus* in a mouse model through NOX-dependent ROS buildup [91].

In addition to the NOX complex, the mitochondrion is a biological source of ROS in immune cells [92]. Once macrophages are activated, the mitochondria favor reverse electron transport in the electron transport chain (ETC), generating ROS [93,94]. Studies have shown that hosts lacking proteins in producing mitochondrial reactive oxygen species (ROS) are vulnerable to infections caused by *Salmonella* Typhimurium [95,96]. For example, mitochondrial ROS were produced and transported directly to the phagosome by mitochondrial-derived vesicles during methicillin-resistant *S. aureus* (MRSA) infection [94]. Additionally, NOX-mediated ROS was observed to influence the antimicrobial capabilities of innate immune cells in vitro and in vivo by modulating the production of inflammatory cytokines, such as interleukin-1 (IL-1) [[97], [98], [99]]. These inflammatory cytokines play a crucial role in orchestrating the immune response, promoting the recruitment and activation of immune cells to the site of infection, and enhancing the overall antimicrobial defense [99].

## 7 The significance of one health in disinfection and zoonotic disease management

The One Health concept emphasizes the interplay among human, animal, and environmental health factors, coupled with the fact that the well-being of one domain likely impacts the other components [99]. Treating zoonotic infections which can spread between animals and humans through direct contact or environmental contamination requires a multidisciplinary approach [100]. The One Health concept emphasizes the necessity for comprehensive solutions to prevent and control dissemination of *Salmonella*, spp. often identified with animal husbandry and food production [101]. One Health provides a holistic approach for delineating the transmission of pathogens across ecosystems, with the ultimate outcome of safeguarding public health, sustaining animal health, and retaining environmental sustainability [100]. Antimicrobial resistance (AMR) in zoonotic non-typhoidal *Salmonella* serovars has arisen as a substantial public health issue, with the use of antibiotics in both human and animal settings exacerbating this worldwide concern [102]. In several contexts, the excessive or improper use of disinfectants may foster the emergence of resistant bacterial strains, which not only endure but also flourish in treated environments, presenting health hazards to both people and animals. The One Health concept promotes a sustainable and integrated strategy for applying disinfectant use across various contexts [103]. One Health approaches offer the opportunity to collaborate across human healthcare, veterinary medicine, and environmental research efforts with the potential to achieve insights into identifying optimal practices that minimize resistance occurrence and retain maximum disinfectant efficacy [103].

Reactive oxygen species produced during exposure to chlorine are particularly efficacious in regulating bacterial populations, including *Salmonella* [104]. The bactericidal effect may be essential for successfully mitigating *Salmonella* infection in environments impacting both animal and human health, including farms, food processing plants, and water systems. In the context of One Health, chlorine-based disinfection that involves ROS represents an opportunity to lower the risk of zoonotic diseases by reducing pathogen populations in public places. Chlorine disinfection could decrease the danger of zoonotic disease transmission by minimizing bacterial pathogens at their origins, therefore offering healthier and safer settings for humans, animals, and their respective ecosystems. Implementing efficient disinfection methods that engage ROS production could markedly reduce cross-species transmission of *Salmonella*, thereby safeguarding human health and promoting animal wellbeing. The One Health strategy encourages a more strategic application of disinfectants to better control pathogenic bacteria [100]. This potentially halts the spread of zoonotic diseases and potentially limits antimicrobial resistance [102]. For example, utilizing suitable doses of chlorine and maintaining optimum contact durations may achieve successful pathogen control without maintaining antimicrobial resistance. This dynamic equilibrium is essential for preserving the sustained effectiveness of disinfectants, safeguarding public health, and improving food safety within interrelated ecosystems.

## 8 Conclusions

*Salmonella* remains a highly prevalent pathogen that severely impacts public health and the poultry industry. Even after years of research on mitigation strategies for limiting this pathogen, the need for development of more effective control measures remains. A combined approach to using disinfectants could help to reduce *Salmonella* contamination of food and agricultural environments while also ensuring long-lasting and more effective cleaning methods that protect public health. Mechanistically, antimicrobials such as chlorine and antibiotics generate ROS, killing bacterial cells by interfering with essential molecules for cellular functions. It has become evident that optimum concentrations of antimicrobials are required to generate enough ROS to kill bacteria effectively. Suboptimal concentrations of antimicrobials can generate less ROS, which leads to the accumulation of mutations that enable subpopulations to become resistant to those antimicrobials and select for dominance of such traits. Further research is needed to understand the mechanisms associated with ROS generation along with the conditions and factors that optimize its production in the presence of *Salmonella*. Understanding these mechanisms could lead to practical approaches for enhancing chlorine disinfectant efficacy. Achieving this understanding supports the One Health concept for managing disinfectants such as chlorine because it can contribute to the interrelated effects on human, animal, and environmental health. Integrating One Health concepts into disinfection procedures may diminish zoonotic disease transmission and alleviate the development of antibiotic resistance.

## Funding

The authors extend their appreciation to the Deanship of Scientific Research at Northern Bordr University, Arar, Saudi Arabia, for funding this research work through project number NBUFFR-137-01.

## CRediT authorship contribution statement

**Mohammed Aljuwayd:** Writing – review & editing, Writing – original draft, Conceptualization. **Israa Abdullah Malli:** Writing – review & editing. **Elena G. Olson:** Writing – review & editing, Validation. **Steven C. Ricke:** Writing – review & editing. **Michael J. Rothrock:** Writing – review & editing. **Young Min Kwon:** Writing – review & editing, Validation, Supervision, Resources, Project administration, Funding acquisition, Conceptualization.

## Declaration of competing interest

None.

## Data Availability

No data was used for the research described in the article.
